# A Focal “Inferno Sign” With an Aberrant Innominate Artery Feeder: A Thyroid Arteriovenous Malformation Mimicking Malignancy in a Young Woman

**DOI:** 10.1155/crie/6016666

**Published:** 2026-11-15

**Authors:** Ampuriire Nyakubaho, Brendah Ayikoru, Monica Acen Okwir, Juliet Nyakake, Joseph Ochom, Deborah Babirye

**Affiliations:** ^1^ Department of Radiology Ernest Cook University (ECU) Kampala Uganda; ^2^ Department of Anatomy Ernest Cook University (ECU) Kampala Uganda; ^3^ Department of Radiology and Radiotherapy School of Medicine Makerere University Kampala Uganda mak.ac.ug; ^4^ Department of Radiology Entebbe Regional Referral Hospital Entebbe Uganda

**Keywords:** color Doppler ultrasound, computed tomography angiography, innominate artery, thyroid arteriovenous malformation, thyroidea ima artery, vascular anomaly

## Abstract

Arteriovenous malformations (AVMs) of the thyroid gland are exceptionally rare, with only 11 cases identified in our review of the literature. They are clinically important because their hypervascularity on imaging can raise suspicion of malignancy, and biopsy or surgery may provoke life‐threatening hemorrhage. We present a case of a 30‐year‐old female with a decade‐long history of anterior neck swelling that recently became painful and enlarged. Ultrasound demonstrated a well‐defined hypoechoic right‐lobe nodule with marked color Doppler hypervascularity (described here as a focal “inferno sign”), a tangled vascular nidus, low‐resistance arterial flow in the dominant feeding vessel, and early arterialization of the draining vein; together, these findings suggested arteriovenous shunting. Computed tomography angiography (CTA) characterized the lesion as a 1.0 cm × 0.9 cm intensely enhancing nodule supplied by an aberrant artery arising directly from the innominate artery, immediately medial to the origin of the right common carotid artery, with drainage into the right internal jugular vein. A radiological diagnosis of thyroid AVM was made without histopathological or angiographic confirmation, and fine‐needle aspiration (FNA) was avoided because of the bleeding risk. The patient was referred for multidisciplinary management but was lost to follow‐up. To our knowledge, this is the first reported thyroid AVM supplied by an innominate artery branch, a variant relevant to surgical and endovascular planning. Thyroid AVM should be considered in the differential diagnosis of hypervascular thyroid nodules, and CTA is valuable for mapping its vascular anatomy noninvasively.

## 1. Introduction

The discovery of a hypervascular thyroid nodule on ultrasound often initiates a diagnostic cascade weighted toward malignancy. Thyroid nodules are detected in up to 68% of the general population on high‐resolution ultrasound, with ~5%–15% proving malignant [1]. The sonographic features that raise suspicion for papillary thyroid carcinoma, that is, marked hypervascularity, hypoechogenicity, irregular margins, and microcalcifications, are, however, not specific to neoplasia. Among the rare benign mimics, arteriovenous malformations (AVMs) occupy an exceptional position, characterized by direct communication between arteries and veins without an intervening capillary bed, resulting in high‐flow shunting that produces dramatic vascularity on imaging [2].

Thyroid AVMs are exceedingly rare; our review of the literature identified only 11 previously reported cases [3–8]. The first description emerged in 2007 when Lee and colleagues [3] identified a thyroid AVM in a patient with Wyburn‐Mason syndrome. Subsequently, Lizarralde and Diaz‐Cano [4] reported two cases in 2013, highlighting the histopathological features and demonstrating the upregulation of VEGF‐A, PDGF‐A, PDGF‐B, and eNOS within these lesions. Černá et al. [5] described a 64‐year‐old man with mechanical neck syndrome from a large (80 mm × 70 mm × 55 mm) thyroid AVM requiring preoperative embolization and partial sternotomy. Borchert et al. [6] documented recurrent high‐flow AVM of the thyroid presenting with dysphagia and dyspnea. Most recently, Almalki et al. [7] reported a symptomatic thyroid/parathyroid AVM in a 32‐year‐old male treated successfully with Onyx embolization, while Goto et al. [8] described the first thyroid AVM in hereditary hemorrhagic telangiectasia (HHT), advocating for 3D‐computed tomography angiography (CTA) as a noninvasive diagnostic substitute for digital subtraction angiography (DSA).

In most prior reports, the diagnosis was established after surgical resection or invasive angiography. In the present case, a radiological diagnosis was reached noninvasively with ultrasound and CTA, although it was not confirmed histologically or angiographically. The feeding vessel arose directly from the innominate artery, immediately medial to the origin of the right common carotid artery, a pattern of supply that, to our knowledge, has not previously been described for a thyroid AVM and that is relevant to interventional and surgical planning. We also use the term focal “inferno sign” descriptively for the marked color Doppler hypervascularity of the nodule; this term has not previously been established for thyroid AVMs, and its diagnostic value here lies in its combination with a vascular nidus, low‐resistance arterial flow, and early venous arterialization.

## 2. Case Presentation

A 30‐year‐old female presented with a 10‐year history of anterior neck swelling that had recently become painful and increased in size. She reported no dysphagia, dyspnea, hoarseness, or symptoms of thyroid dysfunction. Thyroid function tests (TSH, free T4, and free T3) were reviewed by the authors at presentation and were within the laboratory reference ranges; the exact numerical values could not be retrieved for this report because the patient was lost to follow‐up. Antithyroglobulin and antithyroid peroxidase antibody results were not available. There was no personal or family history of HHT, vascular malformations, or thyroid disease. Physical examination revealed a palpable, nontender nodule in the right thyroid lobe without an overlying bruit or thrill. The neck swelling (goiter) was attributable to this solitary right‐lobe nodule; the left lobe was normal on imaging, and no other cause, such as diffuse or multinodular enlargement, was identified. The iodine status was not assessed. The cause of the recent painful enlargement could not be established; progressive shunt‐related vascular dilatation or intralesional hemorrhage were possible explanations.

### 2.1. Ultrasound Findings

High‐resolution ultrasound of the thyroid gland (Figure 1) demonstrated a well‐defined hypoechoic nodule measuring 1.16 cm × 0.72 cm in the right lobe. The nodule exhibited posterior acoustic enhancement on B‐mode imaging. Color Doppler interrogation revealed intense hypervascularity, with a diffuse, confluent color signal filling the entire nodule, which we describe as a focal “inferno sign” (Figure 1). Within the lesion, a focal, tangled “nidus” of vessels was identifiable. Spectral Doppler analysis of the dominant feeding vessel demonstrated a low‐resistance flow pattern (resistive index < 0.5) (Figure 2), consistent with arteriovenous shunting. Early systolic arterialization of the draining vein was also observed, suggesting direct arteriovenous communication bypassing the capillary bed. Ultrasound did not demonstrate a direct connection between the lesion and a cervical artery; the origin of the feeding vessel was identified only on CTA.

**Figure 1:**
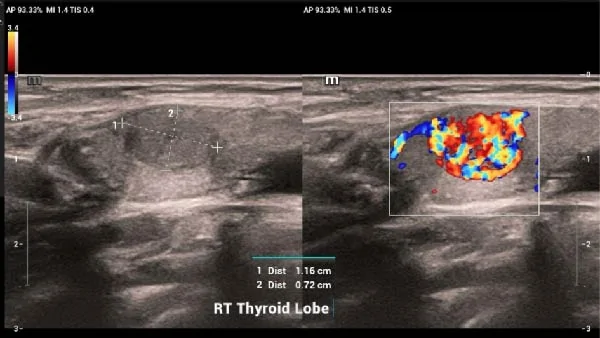
Transverse high‐resolution ultrasound of the right thyroid lobe. Left panel (B‐mode): well‐defined hypoechoic nodule (calipers, 1.16 cm × 0.72 cm) with posterior acoustic enhancement. Right panel (color Doppler): intense, confluent intranodular color signal filling the nodule, described in this report as a focal “inferno sign.”

**Figure 2:**
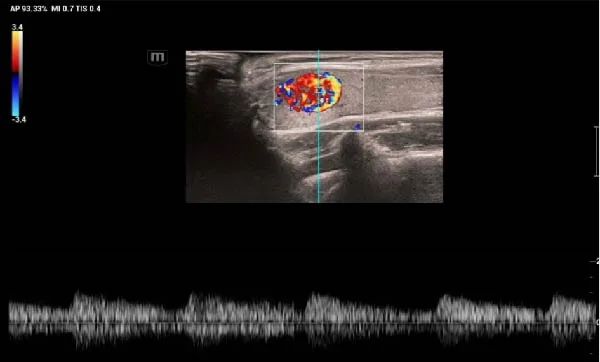
Color and spectral Doppler ultrasound of the right thyroid nodule. Upper panel: color Doppler image showing the tangled vascular “nidus” within the nodule, with the pulsed‐wave Doppler cursor (vertical line) placed across the dominant feeding vessel. Lower panel: corresponding spectral waveform showing continuous forward flow throughout diastole with low pulsatility (resistive index < 0.5), a low‐resistance pattern consistent with arteriovenous shunting. The horizontal axis represents time, and the vertical axis represents flow velocity (cm/s).

### 2.2. CTA

CTA of the neck vessels with multiplanar reformats was performed to characterize the vascular anatomy. A 1.0 cm × 0.9 cm nodule was identified in the right thyroid lobe (Figure 3), with an attenuation of 70 Hounsfield units (HUs) measured in the arterial phase. The nodule exhibited marked heterogeneous enhancement in the arterial phase, mild heterogeneous enhancement in the venous phase, and became isodense to the normal thyroid parenchyma in the delayed phase, a wash‐in/wash‐out pattern typical of high‐flow vascular lesions. The feeding artery was a moderately sized vessel (maximum diameter 0.3 cm and length ~4.9 cm) arising from the innominate artery immediately medial to the origin of the right common carotid artery (Figure 4). It coursed parallel to the right common carotid artery before entering the nidus (Figure 4). Venous drainage occurred directly into the right internal jugular vein (Figure 5). The left thyroid lobe and all major cervical vessels demonstrated a normal anatomy.

**Figure 3:**
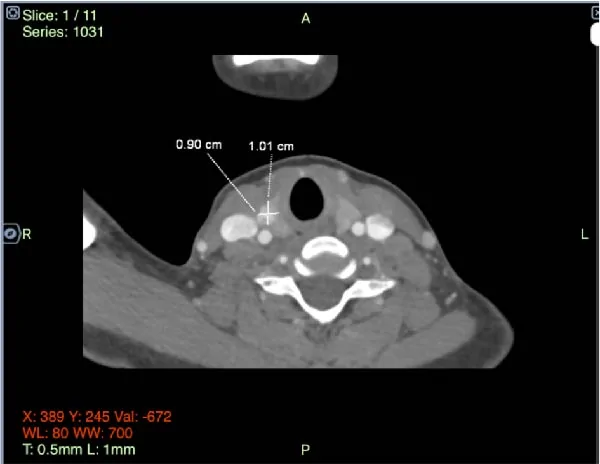
Axial contrast‐enhanced CT angiography image of the neck showing an enhancing 1.0 cm × 0.9 cm nodule (calipers) in the right thyroid lobe, adjacent to the trachea.

**Figure 4:**
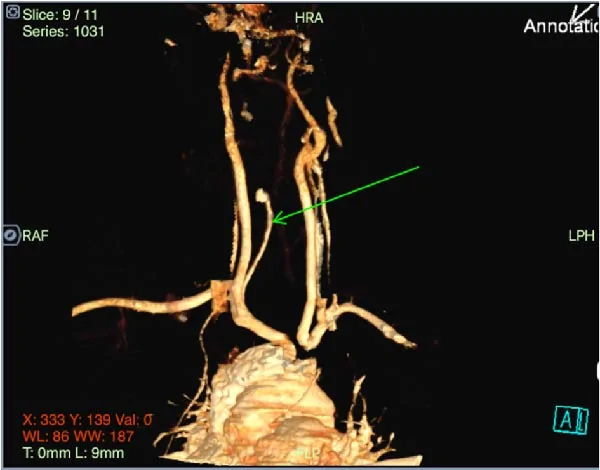
Three‐dimensional volume‐rendered CT angiography reconstruction of the neck arteries (anterior view) showing the feeding artery (green arrow) arising from the innominate artery immediately medial to the origin of the right common carotid artery and coursing upward, parallel to the right common carotid artery, toward the right thyroid lobe.

**Figure 5:**
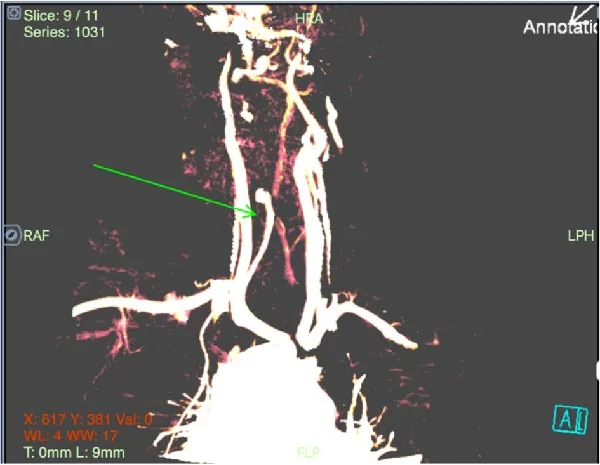
Narrow‐window three‐dimensional CT angiography reconstruction of the neck vessels showing a small draining vessel (green arrow) from the lesion emptying directly into the right internal jugular vein.

### 2.3. Imaging Impression and Clinical Course

The imaging findings were strongly suggestive of an AVM of the right thyroid lobe, supplied by an aberrant branch of the innominate artery with drainage into the right internal jugular vein. Fine‐needle aspiration (FNA) was deferred because of the bleeding risk. The patient was referred for multidisciplinary discussion involving interventional radiology and endocrine surgery to decide between observation with serial imaging, diagnostic angiography with possible preoperative embolization, and surgical resection, but she was lost to follow‐up before a management decision was made.

## 3. Discussion

### 3.1. Imaging Features and the Focal Inferno Sign

On B‐mode ultrasound, thyroid AVMs typically appear as well‐defined hypoechoic nodules that overlap with both adenomatous nodules and malignant tumors [4]. In our case, posterior acoustic enhancement (Figure 1), which is unusual in malignancy, and the absence of microcalcifications, irregular margins, or a taller‐than‐wide shape argued against papillary carcinoma, although these features cannot exclude it.

The term “thyroid inferno” was originally used to describe the diffuse hypervascularity of Graves’ disease [9], and it has not been established as a sign of thyroid AVM. We use the “focal inferno sign” descriptively for the confluent color signal confined to the nodule. Marked hypervascularity alone is nonspecific and also occurs in Graves’ disease, thyroiditis, hyperfunctioning adenomas, and some carcinomas. In our case, its diagnostic value arose from its combination with a discrete nidus (the tangled cluster where arteries communicate directly with veins), low‐resistance arterial flow reflecting the absent capillary bed, and early arterialization of the draining vein, that is, pulsatile flow in a vessel expected to show continuous venous flow [10].

On CTA, intense arterial‐phase enhancement, early venous drainage, and washout to isodensity on delayed images supported a high‐flow shunt, whereas thyroid malignancies more often show persistent enhancement. CTA also showed a single feeding artery arising from the innominate artery, which, to our knowledge, has not previously been reported as the arterial supply of a thyroid AVM.

A thyroid‐supplying artery arising from the innominate artery most likely represents a thyroidea ima artery, a variant found in ~3.8% of individuals that arises from the brachiocephalic trunk in about 74% of cases [11]; an accessory inferior thyroid artery is an alternative interpretation. AVMs are generally regarded as congenital errors of vascular development that enlarge over time [2]. We therefore consider it most likely that this vessel existed as an anatomical variant before the AVM became clinically apparent and that its caliber and length increased secondarily because sustained high flow through a low‐resistance shunt causes progressive dilatation, elongation, and tortuosity of feeding arteries. Without earlier imaging, however, the timing cannot be confirmed.

### 3.2. Pathogenesis and Endocrine Considerations

The pathogenesis of thyroid AVMs is incompletely understood. In previously reported tissue, Lizarralde and Diaz‐Cano [4] demonstrated upregulation of VEGF‐A, PDGF‐A, PDGF‐B, and eNOS in AVM‐associated adenomatous nodules compared with normal thyroid tissue and benign controls.

Goto et al. [8] described a thyroid AVM in a patient with HHT, chronic thyroiditis, and hypothyroidism and suggested that inflammation and TSH elevation may have provided a “second hit” in the setting of a germline mutation. Any role for TSH in sporadic thyroid AVMs remains a hypothesis; in our patient, thyroid function was normal, and no tissue or molecular analysis was available to test any mechanism.

Because AVMs are vascular rather than parenchymal lesions, normal thyroid function is expected; where dysfunction has been reported, as in the patient of Goto et al. [8], it has been attributed to coexisting thyroid disease rather than to the AVM itself.

### 3.3. Management Implications

Management options for thyroid AVMs include observation, embolization, surgery, or a combination, guided by symptoms, lesion size, angioarchitecture, and patient preference. Observation with serial imaging may be reasonable for small asymptomatic lesions, but the natural history is poorly characterized, and enlargement, symptom development, and hemorrhage remain concerns [5, 6].

Preoperative embolization can reduce operative blood loss in large or complex lesions; Černá et al. [5] embolized feeders from the thyrocervical trunk and superior thyroid artery with n‐butyl cyanoacrylate before surgery, and Almalki et al. [7] used Onyx (ethylene vinyl alcohol copolymer) embolization as the primary treatment of a symptomatic thyroid/parathyroid AVM.

DSA remains the reference standard for AVM characterization and embolization guidance, although Goto et al. [8] proposed 3D‐CTA as a noninvasive diagnostic alternative that clearly depicts the feeding artery and draining vein. In our case, CTA provided sufficient anatomical detail for initial planning, including identification of the innominate artery feeder, whose origin and course would influence the choice between embolization and direct surgery; DSA would still be required before any endovascular treatment.

Surgical resection (lobectomy or total thyroidectomy) is the definitive treatment for symptomatic thyroid AVMs, but intraoperative bleeding can be substantial; Ajadi et al. [10] reported an estimated blood loss of 2 L when an unsuspected thyroid AVM was encountered during lobectomy. This underscores the value of preoperative recognition. In our case, the small size (1.0 cm) and well‐defined anatomy might have permitted resection without embolization, but this decision required a multidisciplinary review.

### 3.4. Biopsy Risk and Syndromic Associations

Misdiagnosis of a thyroid AVM as malignancy can lead to FNA, which carries a risk of bleeding and usually yields blood with scant diagnostic material [4]. Both patients reported by Lizarralde and Diaz‐Cano [4] had multiple nondiagnostic FNAs before lobectomy. Radiological recognition is therefore a patient safety issue.

Thyroid AVMs have been reported in Wyburn‐Mason syndrome [3] and in HHT [8]. Our patient had no clinical features of either syndrome, and genetic testing was not performed.

## 4. Limitations

This report has several limitations. First, as a single case, the innominate artery feeder may represent an individual anatomical variant rather than a general feature of thyroid AVMs. Second, the diagnosis is radiological: neither histopathological confirmation nor DSA was obtained, so the imaging findings are strongly suggestive of, but not definitive for, AVM, and the detailed angioarchitecture (e.g., intranidal aneurysms and the exact number of feeders) remains incompletely defined. Third, numerical thyroid function results could not be retrieved, thyroid autoantibody results were not available, and genetic testing was not performed. Finally, the patient was lost to follow‐up, so the outcome, treatment response, and natural history of the lesion are unknown.

### 4.1. Radiology Take‐Home Points

1. Thyroid AVMs are rare but should enter the differential diagnosis for hypervascular thyroid nodules, particularly in young patients without risk factors for thyroid malignancy.
2. Marked color Doppler hypervascularity (a focal “inferno sign”) is not specific on its own; AVM should be suspected when it is combined with a discrete vascular nidus, low‐resistance arterial flow, and early venous arterialization.
3. CTA noninvasively maps the feeding artery and venous drainage; because feeders may arise from unexpected sources, such as the innominate artery in this case, coverage should extend from the aortic arch to the skull base.
4. FNA should be avoided when AVM is suspected, and management should be planned by a multidisciplinary team.

## 5. Conclusion

In this 30‐year‐old woman with long‐standing anterior neck swelling, the combination of ultrasound findings (marked hypervascularity described as a focal inferno sign, a vascular nidus, low‐resistance arterial flow, and early venous arterialization) and CTA features (arterial‐phase hyperenhancement, early venous drainage, and an identifiable feeding artery) was strongly suggestive of a thyroid AVM. This radiological diagnosis, made without histopathological or angiographic confirmation, allowed high‐risk biopsy to be avoided. The feeding vessel arose directly from the innominate artery, a pattern that, to our knowledge, has not previously been described and that underlines the need for comprehensive vascular imaging before surgical or endovascular treatment. Recognizing this benign but potentially hazardous mimic of malignancy is the first step in preventing iatrogenic complications.

## Funding

The authors have nothing to report.

## Disclosure

All authors have read and approved the final version of the manuscript. Ampuriire Nyakubaho had full access to all of the data in this study and takes complete responsibility for the integrity of the data and the accuracy of the data analysis. The authors reviewed all content and take full responsibility for the manuscript.

## Ethics Statement

Local institutional policy did not require ethical approval for this single‐patient case report. This case report was prepared in accordance with the CARE guidelines; the completed CARE checklist is provided as a supplementary file.

## Consent

Written informed consent for the publication of this case report and the accompanying clinical images was obtained from the patient herself, an adult with full decision‐making capacity. The images have been de‐identified. Written informed consent for publication was obtained from the patient as stated above.

## Conflicts of Interest

The authors declare no conflicts of interest.

## Data Availability

The data that support the findings of this study are available upon request from the corresponding author. The data are not publicly available due to privacy or ethical restrictions.
